# Transitional housing in forensic mental health: considering consumer lived experience

**DOI:** 10.1186/s40352-019-0091-z

**Published:** 2019-05-20

**Authors:** Clark Patrick Heard, Jared Scott, Allan Tetzlaff, Heather Lumley

**Affiliations:** 1 Southwest Centre for Forensic Mental Health Care, School of Occupational Therapy, Western University, 401 Sunset Drive, St. Thomas, Ontario N5P 3V9 Canada; 2 Lawson Health Research Institute, London, Canada; 3 St. Leonard’s Community Services, London and Region, 405 Dundas Street, London, Ontario N6B 1V9 Canada

**Keywords:** Transitional housing, Mental health, Forensic, Qualitative method

## Abstract

**Background:**

For individuals involved in the forensic mental health system, access to transitional housing can offer a bridge between custody and independence. Using a methodology consistent with interpretative phenomenological analysis (IPA), this study considers the meaning associated with such participation. In this Canadian study, data was collected via interview with six individuals (*n* = 6) who resided, for a minimum six (6) months, in justice focused transitional housing that involved a partnership between a rural forensic mental health care facility and a nearby urban transitional housing provider.

**Results:**

Following each participant interview, data was transcribed verbatim and coded for themes. Multiple methods were employed to support trustworthiness. Results indicate that participation enabled enhanced social participation, self-esteem/efficacy, community integration and renewal of daily living skills. Participants identified that involvement in justice focused transitional housing enabled development of community living skills, cultivated self-confidence and enhanced personal resilience in their transition from a secure forensic mental health facility to more independent community tenure.

**Conclusions:**

Participants in this research clearly identified the importance of transitional housing programs in supporting their move from a forensic mental health facility to the community. Not all forensic involved individuals will need this type or level of support to support their transition. Practically, however, the nature of forensic hospitalization can present real challenges for occupational participation and maintenance of community living skills. Transitional housing, accountable to unique forensic mental health and justice inputs, can offer a valuable bridge to the community.

## Background

For decades, significant scholarly attention has been devoted to consideration of mental health housing and related community support structures. The practical question of how to optimally support individuals outside of hospital, and with enhanced independence, has been an ongoing question in care. Indeed, in the post de-institutionalization era, supporting individuals to reside in communities and environments of choice has been a key theoretic pillar (Allness & Knoedler, 1998; Anthony, Cohen, & Farkas, 1990; Stein & Santos, 1998). Despite this scholarly attention there remains no consensus on how to best support individuals with serious mental illness in residing in the community.

Over the past several decades, significant scholarly attention has been paid to concepts related to supporting individuals with serious mental illness to reside within their own, more independent, residential settings. The most prominent of these models, Assertive Community Treatment (ACT), can trace its origins directly to the post deinstitutionalization era of the early 1970s. Practically, ACT teams have been designed to support individuals who are not easily engaged in mental health care systems and/or those who more regularly access acute care systems. The mechanisms of such support are an in vivo focus where intensive, long-term, relational and multi-disciplinary teams deliver the services where individuals need to use them (Salyers & Tsemberis, 2007; Ministry of Health and Long Term Care, 2005). To date, this approach has achieved notable primacy in community mental health care provision (Johnson, 2011).

Over time, other models have also come to be considered including concepts of case management, transitional discharge from hospital to community based team support and, in recent years, Housing First initiatives (Forchuk et al., 2013; Kriegel, Henwood, & Gilmer, 2016). Practically, these varied approaches have largely targeted the development of independent living situations while concurrently supporting individuals transitioning from hospital to community.

For individuals who might be ready to leave hospital and return to community living but who may, at least initially, require enhanced support in navigating the day-to-day demands of community living it appears that transitional housing can offer a valuable waypoint. To date, however, consideration of the role of transitional housing in the forensic mental health or broader justice context has been limited and, largely, this work has focused upon institutionally driven narratives and more macro outcome measurement such as concepts of recidivism and length of community tenure (Melnick, 2016; Salem et al., 2015). Practically, this study considers the participant perspective regarding to the role and value of transitional housing as a support for individuals affiliated with the forensic mental health system.

## Review of the literature

Transitional housing supports consumers of mental health services with the opportunity to move from a care facility to the community while maintaining key therapeutic relationships and structural supports (Siskind et al., 2014). Practically, transitional housing acts as a “bridge” between care facility and community (Salem et al., 2015; Barr, Brown, Quinn, McFarlane, McCabe, & Whittington, 2013). In practice, transitional housing supports vary widely in design, structure, potential length of tenure and staffing models (Choy-Brown, Stanhope, Tiderington, & Padgett, 2016). That said, there are some commonalities that define the genre. Typically, this type of housing offers a time limited residential tenure including access to supportive staff/resources with the goal of enabling individuals to move on to more independent living situations. As well, transitional housing generally involves the operationalization of a continuum of care between a sending facility (typically a hospital) and community partners (transitional care provider) with staffing and professional support delivered at the consumer’s place of residence and in other community contexts, as indicated (Vorhies Klodnick, David, Fagan, & Elias, 2014; Yu, 2010).

Transitional housing has been utilized in practice with a wide variety of clinical and non-clinical populations including persons with serious and persistent mental illness, veterans, criminal justice system offenders, and homeless persons (Brown & Wilderson, 2010; Cherner, Aubry, Ecker, Kerman, & Nandlal, 2014; Tsai, Rosenheck, & McGuire, 2012; Yu, 2010). It is difficult to compare various approaches, however, as specific design and operation of transitional housing supports can vary markedly from region to region or in response to the unique populations served. This variance in design and operation makes direct program-to-program comparisons and analysis, even in more macro studies, particularly challenging (Salem et al., 2015). Employing a qualitative phenomenological methodology, this study focuses upon the consumer’s experience of residence in transitional housing within the forensic mental health and justice context. The study accounts the personal narratives and voices of consumers in considering those impacts.

## Methods

### Design

This study employs a qualitative research method consistent with Interpretative Phenomenological Analysis (IPA) to explore the research question: What is the meaning associated with participation in a transitional housing program for forensic mental health consumers (Smith, Flowers, & Larkin, 2009)? Practically, IPA takes an idiographic approach in understanding phenomena in context; “given the complexity of most human phenomena, IPA studies benefit from a concentrated focus on a small number of cases” (Smith et al., 2009, p. 51).

Accordingly, this study focuses on the consumer experience of participation, meaning making, and their perceptions of the value of this participation in care. A phenomenological approach was employed as the study focused upon exploring the unique meaning and experience of participants (Creswell, 2009). In choosing this type of design it is notable that Smith (2011) indicated: “IPA is concerned with the detailed examination of personal lived experience, the meaning of experience to participants, and how participants make sense of that experience” (p. 9). Prior to commencing the study, ethics approval was obtained from The University of Western Ontario (London, Canada) Research Ethics Board (File 106,795). Institutional approval was obtained from Lawson Health Research Institute following Clinical Research Impact Committee review. All participants completed a written consent to participate including consent for potential publication of the research outcomes.

### Participants

For this study, the researchers recruited participants who were affiliated, via Disposition Order from the Ontario Review Board, with the Southwest Centre for Forensic Mental Health Care in St. Thomas, Ontario. Accordingly, these participants, while resident in the community, were legally required by the Ontario Review Board to be supported by the Forensic Outreach Team at the Southwest Centre for Forensic Mental Health Care in the provision of direct community mental health care. To meet inclusion criteria for this study, these same participants were also required to have resided, for at least six months, within a transitional housing setting operated by St. Leonard’s Community Services, London and Region (Gallagher Centre, Madame Louise Arbour Centre, or C. K. Clarke Center).

St. Leonard’s Community Services, London and Region (SLCS), is a community partner of the Southwest Centre for Forensic Mental Health Care and has a mandate that is dedicated to promoting positive change in all persons who are or could be in conflict with the law to realize their potential, contributing to a safer and healthier community (St. Leonard’s Community Services, London and Region, 2017). This differentiates their care model from other transitional housing services in that their focus is in supporting individuals who have come into conflict with the justice system in Ontario, Canada. Their partnership with the Southwest Centre for Forensic Mental Health Care began in 2008 and their Madame Louise Arbour Centre, Gallagher Centre, and C. K. Clarke Center facilities offer secure and controlled entry residential settings with twenty-four hour staffing. This type of care environment supports a highly structured and accountability driven approach for those transitioning to more independent community residence. In some cases, the SLCS secure settings, staffing intensities and focus on provision of care may offer some individuals with their only opportunity for potential community transition. That is, due to supervision restrictions that may be present in an Ontario Review Board Disposition Order, a path to community residence while under the auspices of the forensic mental health system in the Province of Ontario, may only be viable with SLCS level supports.

Participants were recruited by a co-investigator, Jared Scott, Occupational Therapist/Unit Lead with the Forensic Outreach Team at the Southwest Centre for Forensic Mental Health Care. A convenience sample (*n* = 6) met the residence tenure requirement and indicated a willingness to participate in the study. It is notable that requiring such a lengthy tenure at SLCS facilities supported a reasonable limitation in the total number of interview participants and that this is consistent with IPA methodology. Another factor that somewhat limited the potential sample was the periodic occurrence of Absolute Discharge for individuals from the Forensic mental health system by the Ontario Review Board. In those instances, potential participants who were absolutely discharged at their annual Ontario Review Board hearing exited the Forensic mental health system with no obligation to provide future contact information.

Demographically, the interview sample (n = 6) included three males and three females. The average age of the participants was 47 years of age with a range from 36 to 64. The average tenure within the Forensic mental health system in Ontario for each participant was 7.6 years with a range from 3.5 years to 11 years. Diagnoses present among the sample included schizophrenia, schizophrenia (paranoid type) and schizoaffective disorder. Interview participants ranged in length of stay in Forensic mental health care settings (prior to moving to SLCS) from a low of six months to a high of six years with an average of 4.25 years. The average length of stay at SLCS facilities among the sample was one year and eight months with a low of six months and a high of two and one-half years. No payments, incentives or other benefits were provided to any participants.

### Data collection

After consent was obtained and documented a brief interview was undertaken. All interviews occurred during regular working hours and at the research participant’s location of choice. No identifying data or information linking the research to the participant was included on the interview form. Limited demographic information was collected on each interview form regarding each participant:

- What is your age?
- What is your gender?
- How long were you in hospital at The Southwest Centre for Forensic Mental Health Care?
- How long did you reside at a St. Leonard’s Community Services, London and Region facility?
- What is your psychiatric illness diagnosis?

Standardized questions on the interview form included:

- How would you describe your participation in supported housing at St. Leonard’s Community Services, London and Region?
- How do you feel that participation in supported transitional housing at St. Leonard’s Community Services, London and Region influenced your move to community living?
- How has participation in the supported transitional housing program at St. Leonard’s Community Services, London and Region impacted your current community living?

Interview responses were transcribed verbatim immediately following interview completion using word processing software.

### Data analysis

The collected and transcribed data for all six participants was analyzed and coded for themes. The coding team included the principal investigator, a spiritual care professional, and a social worker. The coding process was completed using an editing style of analysis in which the text of each interview was analyzed and meaningful sections identified (Jongbloed, 2000; Krefting, 1991). Each member of the coding team independently analyzed the data and developed preliminary codes. Following this step, the coding team met to identify potential connections and consider the development of overarching themes (Smith et al., 2009). After completion of the data analysis process the final coding scheme was tested for consistency. In this process each coding team member independently coded one transcript using the overarching themes developed in the coding process.

### Trustworthiness

Several methods were utilized to enhance trustworthiness and to reduce the potential for systemic bias. The first of these involved triangulation of data and this involved the collection of data at different times, in different settings and from varied informers. In considering the meaning of the collected data, triangulation by analyst and by theory/perspective was employed (Krefting, 1991; Hammell & Carpenter, 2000). In employing multiple researchers from different health care backgrounds (i.e., occupational therapy, social work, and spiritual care) within the data analysis process, triangulation by theory/perspective was also supported. Finally, both the developed codes and related themes were peer reviewed. Hammell & Carpenter (2000) have noted that this type of review enables “a further instance of triangulation” (p. 111).

## Results

The coding process identified several key thematic categories that described the consumer experience of residence in transitional housing facilities at SLCS in London, Ontario The first of these was in relationship, via the availability and presence of staff in supporting their care. The second was personally, via the opportunity to be supported in personal growth/agency and autonomy. The final area of impact was practical, via opportunities to develop or renew community living skills. The constellation of these three impacts appears to significantly inform the lived experience of study participants.

These summarized themes are described in Table 1.

**Table 1:** Overarching Themes

Relational	
● Relationship and holistic support are key informers	
● An opportunity to enhance quality of life and grow through shared experience	
Personal	
● Participation fostered personal agency (belief, awareness, confidence, esteem, hope)	
● Challenges in adapting to the environment enabled personal growth	
Practical	
● Experiential learning enabled practical and transferrable competency development	
● Participation has cultivated confidence and enabled resilience	

Three interrelated thematic categories of overarching themes describe the impacts of participation in transitional housing settings at SLCS for individuals affiliated with the Southwest Centre for Forensic Mental Health Care. These categories of overarching themes spoke to impacts in care for the participants that were Relational, Personal or Practical.

The first of these impact categories, Relational, identified the centrality of relationship and holistic support in care provision for participants.

Of this, one participant noted:

The supports … the staff being available. If you have questions or concerns or wanted someone to talk to they were always available. They could talk to you, or if you were having trouble with someone they could talk to them, so you never had to be worried and always had someone to help (participant 4).

Other participants similarly spoke to concepts of staff availability noting: “The staff were involved. It was motivating, a little bit. It helps you follow your plan and feel motivated” (participant 5). Another indicated: “It’s been pretty good. I like it here. The people are very cordial. The staff is nice. I have no problems with them” (participant 2).

The opportunity to enhance quality of life through shared experience with program staff and peers was a second overarching theme that spoke strongly to the centrality of relationship within the care paradigm. Clients spoke to the opportunity to take part, to feel included, to contribute and to belong. One participant noted that:

I guess what would really stand out was the meal and recipe planning. I found it outstanding that one person would cook for a lot of other people. It showed a lot of maturity. If you can cook for other people, that’s pretty mature (participant 6).

Another noted that the opportunity to contribute was relevant indicating: “I did chores. It was always great to have support, the door was always open and there were people to talk to” (participant 4). That same participant spoke to participation with staff and peers and how this impacted their transition to community noting: “… they had parties, like Christmas and Halloween, so it made it easier to be away from your families” (participant 4).

The second key thematic category spoke to Personal impacts as a key outcome of participation at SLCS in transitional housing settings. Participants described an experience where challenges in adapting to the environment enabled personal growth. In this context, participants spoke to the concept that adjusting to the transitional care environment, despite difficulty at points, supported their development. Of this, one participant noted: “You had to get along with others, adapt to others. That can be stressful. It’s hard to adapt to the norms of the house” (participant 3). Another noted, simply: “There’s more responsibility here” (participant 2). Adapting to the environment did not come without challenges for the participants, however, and several put voice to this content. One participant noted that at one facility they perceived that: “… there was a lot of distraction. A lot of triggers to do drugs. There were too many people in the living space” (participant 5).

Along with personal growth, participants described how residence at SLCS facilities enabled enhanced personal agency including belief, awareness, confidence, esteem and hope. Participants were plain in their discussion of this content, noting: “It’s given me a chance to put on a good presentation and to allow me to prove myself in a starter home in the community. I think that it’s a good stepping-stone.” (participant 1). Another noted: “Moving to the home meant more independence and it gives you hope with the forensic system sometimes; that you’re doing something right” (participant 5). Speaking to this same content, another contextualized it in consideration of past residence in hospital indicating:

When you’re in the hospital you feel like you’re in the hospital. But when you’re living at St. Leonard’s you feel like part of the community or someone just down the street. It feels like you’re at home. It’s homelike, not a centre or something like that (participant 4).

The final key thematic category spoke to the more practical impacts of participation at SLCS facilities in transitional housing. Participants described an experience in residence where experiential learning enabled practical and transferrable competency development for community living and participation. Participation in transitional housing of this nature for individuals affiliated with a forensic mental health care setting enabled a safe learning environment in which they could take on novel responsibilities and develop or renew skills.

One spoke to undertaking practical skills: “cleaning, cooking, groups, games, shopping, medication dispensing and the support network they have in the home have all been helpful. That’s been awesome. And the support upheld by the hospital” (participant 1). Another noted that: “at the beginning I was nervous about cooking and would need to take a PRN (as needed prescription medication) … but now I’m not nervous and I stopped taking that a long time ago” (participant 2). A third participant also spoke to practical outcomes indicating: “they helped me with budgeting and meal planning. And they helped me find … [a housing program] … because that was my housing and support post St. Leonard’s and that has been a great progression” (participant 6).

In considering community participation, one participant stated: “It helped me introduce myself to businesses, and walking around in the community, and just re-establishing my connections to the community” (participant 6). Another noted:

Here I do social work. I do chores here. We have chores like cooking and leaves, and cleaning and stuff. I do the cooking here, sometimes, for the residents, once or twice a week. I clean the dishes and the dishwasher, do my laundry, go out shopping. I go out on outings, like to the pool to swim. I go to stores to browse and shop. It’s close to lots of good stores for shopping. All these things help me get ready to live outside this house (participant 2).

One participant clearly noted: “Living at St. Leonard’s helped me become more motivated and willing to do stuff and try new things” (participant 5). Not all participants found all elements of experiential learning helpful or relevant, however. One noted: “the groups … were not that helpful to me. It was great to have people to chat with but not that helpful …” (participant 5).

A second practical theme spoke to the concept that transitional housing participation cultivated confidence and enabled resilience. Participants spoke meaningfully to this content noting: “It’s given me a good review and presented me with further insight into my ability to live in the community and increased my capacity to live alone. It’s helped me by providing support. It’s been a good place to integrate me back into society and I think I’ll be successful in my integration to the community because of it. I’m sure I will be” (participant 1). Another noted: “I’m more comfortable with myself. I’m more comfortable living on my own now that I’ve had the practice” (participant 3). A third participant indicted:

Overall it was very good for both places (referencing two of the SLCS care centres). They were able to help me find an apartment. Having support for that time period gave me confidence to live by myself. I had never lived by myself before, I’d always lived with my family (participant 4).

Finally, a participant stated: “I feel like I’m more ready to tackle problems. I have more skills and knowledge. I’d be more ready and know what to do if I got sick. I’m able to control my illness and not allow it to be severe” (participant 5).

Participants did not find all aspects of participation at SLCS facilities to be helpful or relevant to their longer-term community living goals. In describing the lived experience they noted a number of concerns. Some of these were fairly practical in nature: “It’s a little restricting because I’m used to living on my own. And there is more responsibility, because I’ve never had to cook for others before” (participant 1). Similarly, another participant indicated: “Sometimes residents do things in a way that is different than I would do it, but I get used to it …” (participant 2). Other concerns related to the nature of residing with other individuals who had come into conflict with the justice system but outside of the forensic or mental health system. One participant indicated: “I think that if you’re coming from the forensic mental health system, it’s easier to be with people with a mental illness, as opposed to people who are not NCR (Not Criminally Responsible)” (participant 4). Another discussed some stressors related to residing with individuals from non-forensic mental health environments resident at SLCS facilities noting that at times: “… you’re dealing with offenders who have committed serious crimes – some of them recent offenders. And there’s a lot of drugs” (participant 5).

## Discussion

This qualitative study considers the meaning associated with participation in a transitional housing program for forensic mental health consumers. In analysis of the results, the thematic data fell into three broad areas of impact for participants: Relational, Personal and Practical. Participants spoke to the centrality of relationships with SLCS staff and of the importance that this support held for them. Specifically, participants indicated that respect and availability were key to enabling therapeutic relationships with staff. This kind of staff support is a central concept in current literature considering transitional housing in mental health care and, particularly, forensic transitions. In specific, Cherner, Nandlal, Ecker, Aubry, and Pettey (2013) describe staff “openness and availability” as key informers for supporting therapeutic relationships with individuals during transition (p. 171). While more structural in design, Melnick (2016) describes supporting forensic transition in Florida using a system of progressive levels driven by the relationship between consumer and counselor. Compellingly, Petersen, Friis, Haxholm, Nielsen, and Wind (2015) speak to this centrality of relationship in supporting transition in mental health care noting:

… social relations have an impact on recovery and that the role of staff … [is] … to help facilitate this process. Recovery is what individuals do; facilitating recovery is what professionals do, and supporting recovery is what systems and communities do (p. 9).

Participants also spoke to the value of being supported in their own personal growth, and future self-determination. In particular, participants identified that their residing at SLCS in transition from the forensic setting supported enhanced self-belief, awareness, confidence, esteem and hope. This narrative of supporting personal growth is central in current literature considering transitional housing in mental health but, also more broadly, within current mental health literature. Bermingham, Manlick, and Liu (2015) in considering community residential support to mediate homelessness among veterans with serious mental illness identify the importance of supporting the development of self-esteem and accomplishment via success in daily living skills and vocational endeavors. Indeed, current consideration of recovery in the context of mental illness speaks not to the absence of symptoms but, rather, to application of self-determinism/personal agency and the renewal of participation in meaningful occupation (Doroud, Fossey, & Fortune, 2015; Kelly, Lamont, & Brunero, 2010).

The potential to develop and generalize community living skills from SLCS facilities to new community environments was identified as important by participants. Participants perceived practical skill development like budgeting and meal planning as important as they moved on to more independent community living situations. The opportunity to develop applicable community living skills within safe and supportive environments may have been the most important outcome for some participants. Perhaps unsurprisingly, this concept is valued within current community mental health literature.

Recent study by Tan et al. (2018) looking at factors associated with community functioning after outpatient rehabilitation identified what appears a strong correlation between recovery and one’s ability to function in the community. Participants also voiced the relevance of feeling included as participants within their community. Bitter, Roeg, van Nieuwenhuizen, and van Weeghel (2016) identified social inclusion and related challenges that individuals with serious and persistent mental illness may face as important areas for focus in rehabilitation. Compellingly, Finnerty et al. (2015) in examination of those factors that support transition from Assertive Community Treatment teams to less intensive services identified community living/participation “skill sets” as necessary for lower levels of care (p. 92). The focus on such “skill sets” at SLCS can support transition to less intensive care or enhanced independence over time and this was clearly perceived by participants in the study.

It does appear that for individuals who might be ready to leave hospital and return to community living but who may, at least initially, require enhanced support in navigating the day-to-day demands that transitional housing can offer a valuable waypoint. This waypoint is not, however, without its complexities and complications. Brolin, Brunt, Rask, Syren, and Sandgren (2016) speak to some of these complexities in their work considering life in supported housing for people with serious and persistent mental illness. Specifically, the authors cite the deprivation of self-determination as a serious concern in such facilities and note, not ironically, that these same concerns were present in psychiatric institutional settings. This content, particularly as related to self-determinism was voiced by several participants in the present study. Practically, this is a complex layer in consideration of consumer participation at SLCS in transitioning from forensic facilities where autonomy and self-determination are purposively limited by the Ontario Review Board and related Disposition Orders. That said, concepts of self-determination and how this may be enhanced at SLCS, accounting legal/forensic system responsibilities, appear worthy of consideration.

### Implications for practice

The participants in this study speak to important themes about their personal experience in transitional housing following interaction with the forensic mental health system. Practically, tenure in forensic mental health facilities supports a complicated narrative in consideration of rehabilitation and the practicality of development/maintenance of community living skills (Craik et al., 2010). That is, supporting or enhancing community living skills while concurrently balancing the intersection of consumer illness experience and facility safety/risk management is a complicated endeavor. Further confounding matters, access to more typical occupations like banking, shopping, or meal preparation may be limited by many factors including an individual’s latent skill/ability, illness experience/impacts, care facility security considerations or simply the ability to access community environments while in hospital. Finally, typical demands of day-to-day living are mediated, compassionately, within hospital facilities as they provide food, medicines and accommodation in order to support recovery. The intersection of all of these factors, however, present very real challenges for rehabilitation professionals within forensic mental health settings. Craik et al. (2010) refer to this situation as an “impoverished occupational environment” and advocate that the “splitting off of occupation to designated areas and times, while fitting with safeguarding and containment agendas compromises the health of all” (p. 343).

Perhaps then, this may be the key space that justice focused transitional care facilities can occupy within the forensic mental health context. That is, they can offer a middle ground between the legally mandated security required within forensic mental health care facilities and the occupational participation capacity required to thrive in community living. Certainly, occupational and community living skills must remain a priority in hospital but these can only be truly tested in immersive community environments. Lindstedt, Grann, and Söderlund (2011) identify that “Forensic patients need intensive, extensive, and intrusive treatment programmes”(p. 309). There is a real opportunity for transitional care facilities to support this intensity of care and enhance the development of resilience and related protective factors that are key for successful transition (Barr et al., 2013; Viljoen, Nicholls, Greaves, Ruiter, & Brink, 2011).

### Limitations and directions for future research

This study does have some limitations. Principally, generalizability may be somewhat limited by the sample size and singular community considered. Practically, this represents something of a compromise for the research team given the required tenure at SLCS transitional care facilities for eligible participants was quite significant. On one hand this tenure requirement limited the total number of potential participants but, on the other, this likely enhanced the richness of the data given that the study focused upon consumer lived experience. Indeed, Smith et al. (2009) have identified that sampling methods for Interpretative Phenomenological Analysis studies should be more or less homogeneous and purposive.

Future research considering the relationship between transitional housing and forensic mental health might reasonably target several areas. The first of these may consider what consumers identify to be the most optimal preparation for participation within such settings. This could have a very real impact on current rehabilitation approaches across a number of disciplines. As well, consumer identification of those factors that support or limit tenure within transitional housing facilities may also be of real value for both the housing providers and sending facilities.

## Conclusion

This qualitative research has considered the lived experience of six individuals participating in transitional housing within a forensic mental health context. Their narratives speak powerfully to the relevance of these types of programs in supporting transition from hospital to community. Practically, not every individual involved in the forensic mental health system will require this intensity of support in transitioning to the community. Many individuals may not require the sort of intensity of care, accountability or structure involved with such a transitional housing setting. However, the nature of forensic hospitalization can present real challenges for occupational participation and maintenance/growth in community living skills and for those who require support in such areas transitional housing with a justice focus can be a powerful resource.

Graffam, Shinkfield, Lavelle, and McPhearson (2004) identify six domains influencing community reintegration for individuals who have come into conflict with the criminal justice system. These include: personal conditions, social network/environment, accommodation, interaction with the criminal justice system, rehabilitation/counseling and employment/vocational potential and outcomes. It is compelling that each of these areas were identified by the participants in this study. This speaks to the real potentials for transitional housing in the mental health and justice context.

## Abbreviations

ACT: Assertive Community Treatment
IPA: Interpretative Phenomenological Analysis
PACT: Program for Assertive Community Treatment
PRN: As needed prescription medication
SLCS: St. Leonard’s Community Services (London and Region)
